# Supplementary material for: A Critical View of the Application of the APEX Software (Aqueous Photochemistry of Environmentally-Occurring Xenobiotics) to Predict Photoreaction Kinetics in Surface Freshwaters
Source: Molecules. 2019 Dec 18;25(1):9. doi: 10.3390/molecules25010009 (PMC7017383; doi:10.3390/molecules25010009)
Supplement: Supplementary file 1 [file molecules-25-00009-s001.zip › 3.2.4_gcc-4.4.0.zip!/3.2.4_gcc-4.4.0/3.2.4_gcc-4.4.0/doc/octave/html/interpreter/Allocating-Local-Memory-in-Oct_002dFiles.html]

Allocating Local Memory in Oct-Files - Untitled


Next: Input Parameter Checking in Oct-Files,
Previous: Calling External Code from Oct-Files,
Up: Oct-Files


---

#### A.1.10 Allocating Local Memory in Oct-Files

Allocating memory within an oct-file might seem easy as the C++
new/delete operators can be used. However, in that case care must be
taken to avoid memory leaks. The preferred manner in which to allocate
memory for use locally is to use the `OCTAVE_LOCAL_BUFFER` macro.
An example of its use is

```
     OCTAVE_LOCAL_BUFFER (double, tmp, len)
```

that returns a pointer `tmp` of type `double *` of length
`len`.
